# Interaction of predatory macroinvertebrate communities with malaria vectors in aquatic habitats of three climatic zones in Burkina Faso

**DOI:** 10.1186/s13071-025-06794-z

**Published:** 2025-04-27

**Authors:** Judicael Ouedraogo, Simon P. Sawadogo, Abdoulaye Niang, Abdoulaye Soulama, Sylvie Yerbanga, Tarwendpanga F. X. Ouédraogo, Bouraïma Vincent Séré, Charles Guissou, Roch K. Dabiré, Ruth Müller, Olivier Gnankine, Abdoulaye Diabaté

**Affiliations:** 1 Institut de Recherche en Sciences de La Santé (IRSS), Direction Régionale de L’Ouest (DRO), 399 Avenue de La Liberté, 01 BP 545, Bobo-Dioulasso 01, Burkina Faso; 2 Laboratoire d’Entomologie Fondamentale Et Appliquée (LEFA), Université Joseph KI-ZERBO, 03 BP 7021 Ouagadougou, Burkina Faso; 3 Laboratoire d’Ecologie Vectorielle Et Parasitaire, Département de Biologie Animale, Université Cheikh Anta Diop, Dakar-Fann, Dakar, BP 5005 Sénégal; 4 Université Nazi Boni, 01 BP 1091 Bobo-Dioulasso 01, Burkina Faso; 5 Unit Entomology, Institute of Tropical Medicine, Nationalestraat 155, Antwerp, Belgium

**Keywords:** *Anopheles* larvae, Predation, Macroinvertebrates, Competition, Climatic zones, Burkina Faso

## Abstract

**Background:**

In aquatic larval habitats, *Anopheles* larvae are subject to the predatory activity and competition of macroinvertebrates. These macroinvertebrates may play a key role in the *Anopheles* population’s bioregulation in aquatic habitats and malaria control. There are few studies characterizing macroinvertebrate predators and other macroinvertebrates coexisting with *Anopheles* larvae in Burkina Faso. This study aimed at characterizing and evaluating the different interactions between anopheline mosquito larvae, predatory macroinvertebrates, and other co-habitants in aquatic habitats in the three climatic zones of Burkina Faso.

**Methods:**

A larval survey was performed in the three climatic zones of Burkina Faso (Sahelian, Soudano-Sahelian, and Soudanian zones) from September to November 2022. Mosquito larvae and other macroinvertebrates were sampled using standard dippers or bucket, preserved in Falcon tubes containing 80% ethanol, and transported to the laboratory for morphological identification. Alpha diversity analysis was used to measure macroinvertebrate diversity according to climatic zones and correlation matrix analysis was performed to determine the different interactions between *Anopheles* and other macroinvertebrates in breeding sites.

**Results:**

In the studied larval habitats*, Anopheles* were found with several aquatic macroinvertebrate predators and other cohabiting macroinvertebrates. The abundance and alpha diversity indices of macroinvertebrate predators and other coexisting macroinvertebrates varied significantly according to climatic zone (*P* = 0.01). Correlation analyses showed that in the Sahelian zone, *Anopheles* spp., Corixidae, and Notonectidae shared the same aquatic habitats. In the Soudano-Sahelian zone, *Anopheles* spp. occupied the same larval habitats with Belostomatidae, Notonectidae, and Achatinidae, and in the Soudanian zone, their presence in larval habitats was correlated with that of Beatidae.

**Conclusions:**

This study showed a significant trophic association between *Anopheles* and predatory and other coexisting macroinvertebrates in larval habitats in Burkina Faso. Our study provides insights and thereby opens new avenues in terms of development of biological control against larvae of *Anopheles* populations in Burkina Faso.

**Graphical abstract:**

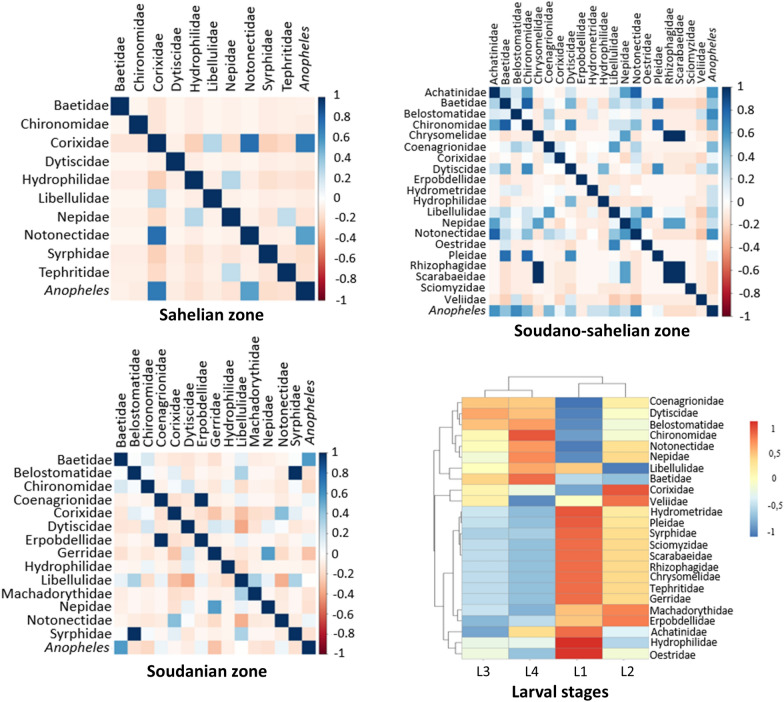

## Background

Up to the present day, mosquitoes remain a threat to human health due to their ability to transmit various infectious diseases such as malaria, one of the deadliest diseases in tropical regions. In many sub-Saharan countries, malaria remains a public health problem [1]. In Burkina Faso, more than 10 million cases of malaria were recorded in 2023 with around 5000 deaths, demonstrating the heavy burden still posed by malaria [2].

Vector control is an essential component of malaria control and elimination strategies. The main interventions that have contributed significantly to reducing the burden of malaria are the use of long-lasting insecticidal nets (LLINs) and indoor residual spraying (IRS). These methods have contributed to reducing significantly the number of malaria cases and deaths worldwide in recent years, but unfortunately the continued spread of insecticide resistance in *Anopheles* mosquitoes threatens the global fight against malaria [3]. Consequently, malaria elimination may not be achieved unless additional tools are found and implemented [4].

Larval control, one of the approaches to vector control, has been neglected thus far in malaria vector control programs [1, 5]. *Anopheles* larvae generally develop in rain-dependent freshwater habitats [6–8]. Studies have shown that high larval mortality is common in natural breeding sites due to several parameters including climatic conditions and predation [9, 10]. In aquatic habitats, anopheline mosquito larvae cohabit with other macroinvertebrates and are susceptible to competition and predation. *Anopheles* larvae and their predators coexist in a variety of aquatic habitats and these predators may contribute to the bioregulation of vector species capable to transmit *Plasmodium* parasites causing malaria disease [11, 12].

The role of aquatic predators in controlling the anopheline mosquito larvae has been known for years, and studies indicate that 90% of the mortality of immature mosquito stages in certain aquatic environments is attributable to predators [13, 14]. The role played by aquatic predators as biocontrol agents in the natural regulation of mosquito larval and adult populations has not been well exploited in vector control. Therefore, there is an urgent need to improve larval source management by considering predatory macroinvertebrates as an evolutionary tool for integrated vector management programmes to reduce vector populations [15–17].

A good understanding of *Anopheles* larval ecology and their interactions with other macroinvertebrates in larval habitats is imperative for malaria control and could inform vector control strategies targeting larval habitats [17]. Studies performed in Burkina Faso showed that the exposure of anopheline mosquito larvae to predators in aquatic environment had an impact on development of larvae, adult size, fecundity, longevity, and choice of larval breeding sites [8, 18, 19]. However, there is little documented data characterizing predators that coexist with *Anopheles* spp. larvae in Burkina Faso. Hence, the aim of this study was to characterize the predatory macroinvertebrates and other coexisting macroinvertebrates associated with *Anopheles* mosquitoes’ larvae in breeding sites in the three climatic zones of Burkina Faso.

## Methods

### Study area

**Fig. 1:**
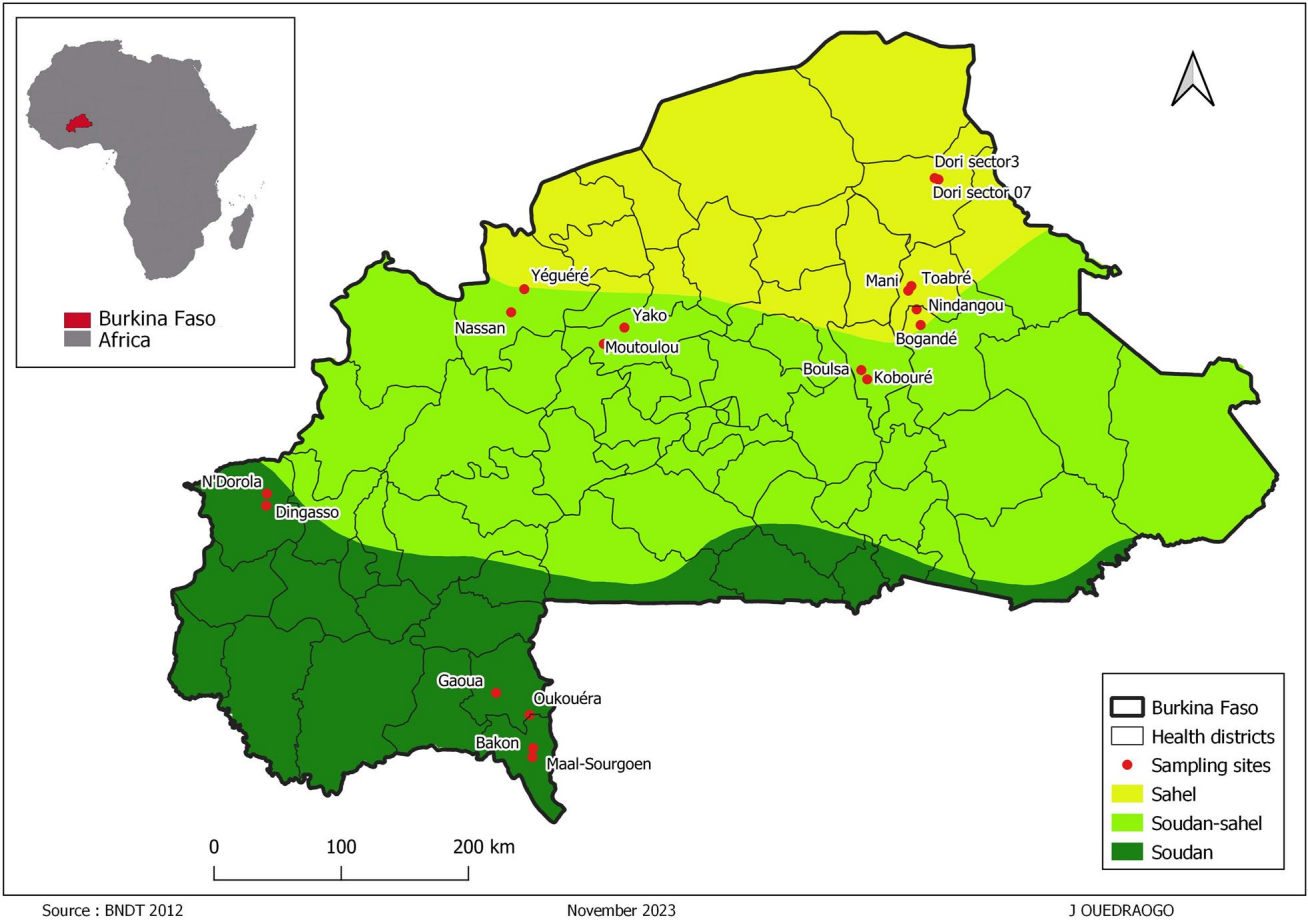
Location of sampling sites (*n* = 18) according to climatic zones in Burkina Faso

This study was carried out in the three climatic zones of Burkina Faso, namely the Sahelian, the Soudano-Sahelian, and the Soudanian zones (Fig. 1). Climate in Burkina Faso is tropical, of the Soudano Sahelian type, characterized by rainfall variations ranging from an average of 350 mm in the north to more than 1000 mm in the southwest. Burkina Faso has two very distinct seasons (rainy season and dry season). The rainy season lasts between 3 and 6 months (May–October) with rainfall ranging from 300 mm to 1200 mm, and dry season lasts around 6 months (November–April) marked by the harmattan, a hot and dry wind blowing from the Sahara. The country is subdivided according to average annual rainfall into three main climatic zones (Fig. 1). The Sahelian climatic zone located in the north part is characterized by rainfall ranging between 300 and 600 mm/year and high temperatures from 15 °C to 45 °C. In the Soudano-Sahelian in the center of country, the annual rainfall varies between 600 and 900 mm/year. The Soudanian zone in the south has a high potential agro-sylvo-pastoral with 900 to 1200 mm/year and relatively low average temperatures [20]. The cumulative rainfall recorded in 2022 at the sampling sites is listed in Table 1.

**Table 1:** Sampling sites [21]

Climatic area	Health region	Geographic references	Sampling sites	Precipitation (mm)
Soudanian zone	N’Dorola	11°46’N; 4°49’W	Dingasso; N’Dorola	1350–1500
	Gaoua	10°20’N; 3°10’W	Gaoua; Oukouéra	1050–1200
	Batié	9°52′60’’N; 2°55’W	Maal-Sourgoen; Bakon	1350–1500
﻿Soudano-sahelian zone	Tougan	13°4’N; 3°4’W	Nassan; Yéguéré	750–900
	Yako	12°57’N; 2°16’W	Moutoulou; Yako	900–1050
	Boulsa	12°39’N; 0°34’W	Boulsa; Kobouré	600–750
Sahelian zone	Bogandé	12°58′1’’N; 0°9’W	Bogandé; Nindangou	600–750
	Mani	13°15′30’’N; 0°12′47’’W	Mani; Toabré	600–750
	Dori	14°1′59’’N; 0°1′59’’W	Dori Sector 3; Dori Sector 7	450–600

### Collection and identification of mosquito larvae and other aquatic macroinvertebrates

Mosquito larvae and other aquatic macroinvertebrates were collected from larval habitats in the three climatic zones of Burkina Faso. Sampling was carried out during the period from September to November 2022 in three health regions per climatic zone. In each health region, two villages were randomly selected as collection sites, and ten larval breeding sites were surveyed per village. A standard dipper (350 ml) and 10 L bucket were used to collect mosquito larvae and other macroinvertebrates from the larval habitats. The mosquito larvae and other macroinvertebrates collected were separated and preserved per larval habitat in 15 ml Falcon tubes containing 80% ethanol. These samples were transported to the laboratory of the Institut de Recherche en Sciences de la Santé/Direction Régionale de l’Ouest (IRSS/DRO). The mosquito larvae were identified by using morphological criteria and counted [22]. The different larval stages of *Anopheles* mosquitoes were determined using sieves (Fisher Scientific Ltd., UK) to separate the larvae according to their size. The other macroinvertebrates were identified on a binocular magnifying glass by using morphological identification keys of Gerber and Gabriel [23], Gill [24], Dejoux et al. [25], Theischinger and Endersby[26], Andersen and Weir [27], Laurince et al. [28], and Tinerella [29]. After morphological identification of the other macroinvertebrates, a literature review was performed to identify the macroinvertebrates that feed on *Anopheles* larvae [1, 30–33]. The group of macroinvertebrates that consume *Anopheles* larvae are the predators. Other macroinvertebrates that do not feed *Anopheles* larvae are considered to be the coexisting macroinvertebrates because they share with anopheline mosquitoes the same larval habitats and food resources.

### Statistical analysis

The data were analyzed using R software (version 4.3.2). The Shannon–Wiener index and Simpson diversity index were calculated using Eqs. 1 and 2, respectively, to determine the alpha diversity of the macroinvertebrate predators and other coexisting macroinvertebrates associated with *Anopheles* larvae in breeding sites in different climatic zones [34]. Heatmaps were performed to assess the different interactions between anopheline mosquito larvae and other macroinvertebrates in aquatic habitats. The Kruskal–Wallis test was used to compare the abundance and alpha diversity index of predatory and other coexisting macroinvertebrates in aquatic environments in the different climatic zones.

1 $$H^{\prime } {\mkern 1mu} = {\mkern 1mu} - \sum\nolimits_{{i = 1}}^{S} {p_{i} {\text{ ln}}\,p_{i} } \,{\mkern 1mu}$$

2 $$D\, = \,1\,\frac{{\sum n\left( {n - 1} \right)}}{{N\left( {N - 1} \right)}}$$

where:

*H*^’^ = Shannon-Wiener index.

*p*_*i*_ = proportion of individuals belonging to species *i.*

ln = natural log.

*D* = Simpson’s diversity index.

*n* = total number of organisms of a particular species.

*N* = total number of organisms of all species.

## Results

### Abundance of predatory and other coexisting macroinvertebrates in aquatic habitats

**Fig. 2:**
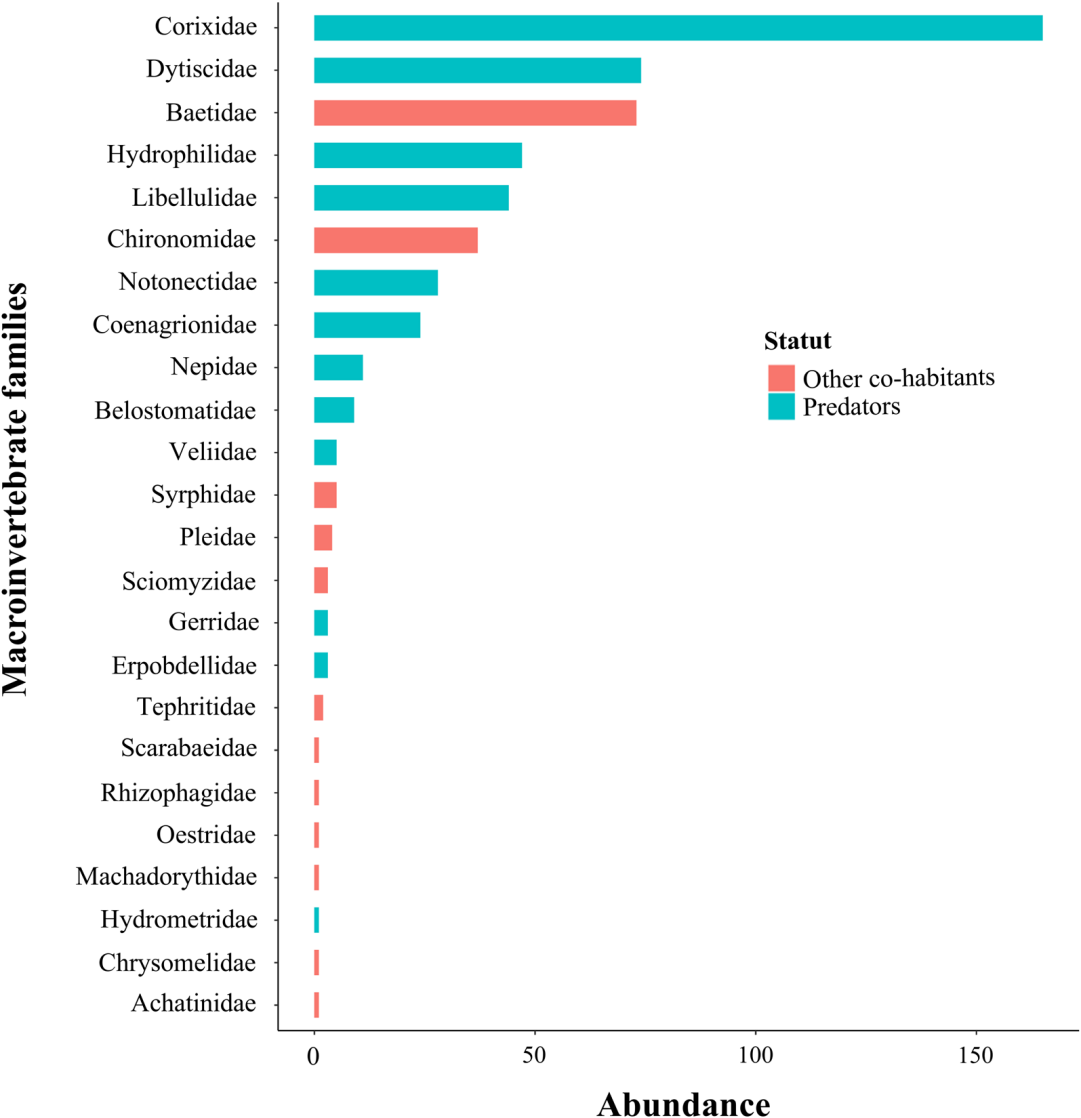
Distribution of predatory macroinvertebrates and other macroinvertebrates coexisting with *Anopheles* larvae

A total of 10,885 macroinvertebrates were collected in mosquito breeding sites in the three climatic zones of Burkina Faso between September and November 2022. Of the total 10,885 macro-invertebrates collected, 10,341 (95%) were mosquito larvae and 544 (5%) were predatory macroinvertebrates and other macroinvertebrates coexisting with *Anopheles* spp. larvae. In this study, of all samples collected, 24 families of predatory macroinvertebrates and other coexisting macroinvertebrates were identified. The ten most abundant families of which in all climatic zones of Burkina Faso were Corixidae (30.3%), Dytiscidae (19.6%), Baetidae (13.4%), Hydrophilidae (8.6%), Libellulidae (7.9%), Chironomidae (6.8%), Notonectidae (5.2%), Coenagrionidae (4.4%), Nepidae (2%), and Belostomatidae (1.7%). Figure 2 summarizes the different macroinvertebrate distribution by family as a function of abundance. Among the predators, the most abundant were Corixidae, Dysticidae, Hydrophilidae, and Libellulidae (Fig. 2). The most abundant of other macroinvertebrates coexisting with malaria vectors were the Baetidae, Chironomidae, Syrphidae, and Pleidae (Fig. 2).

### Distribution of predatory and other coexisting macroinvertebrates according to climatic zones

**Fig. 3:**
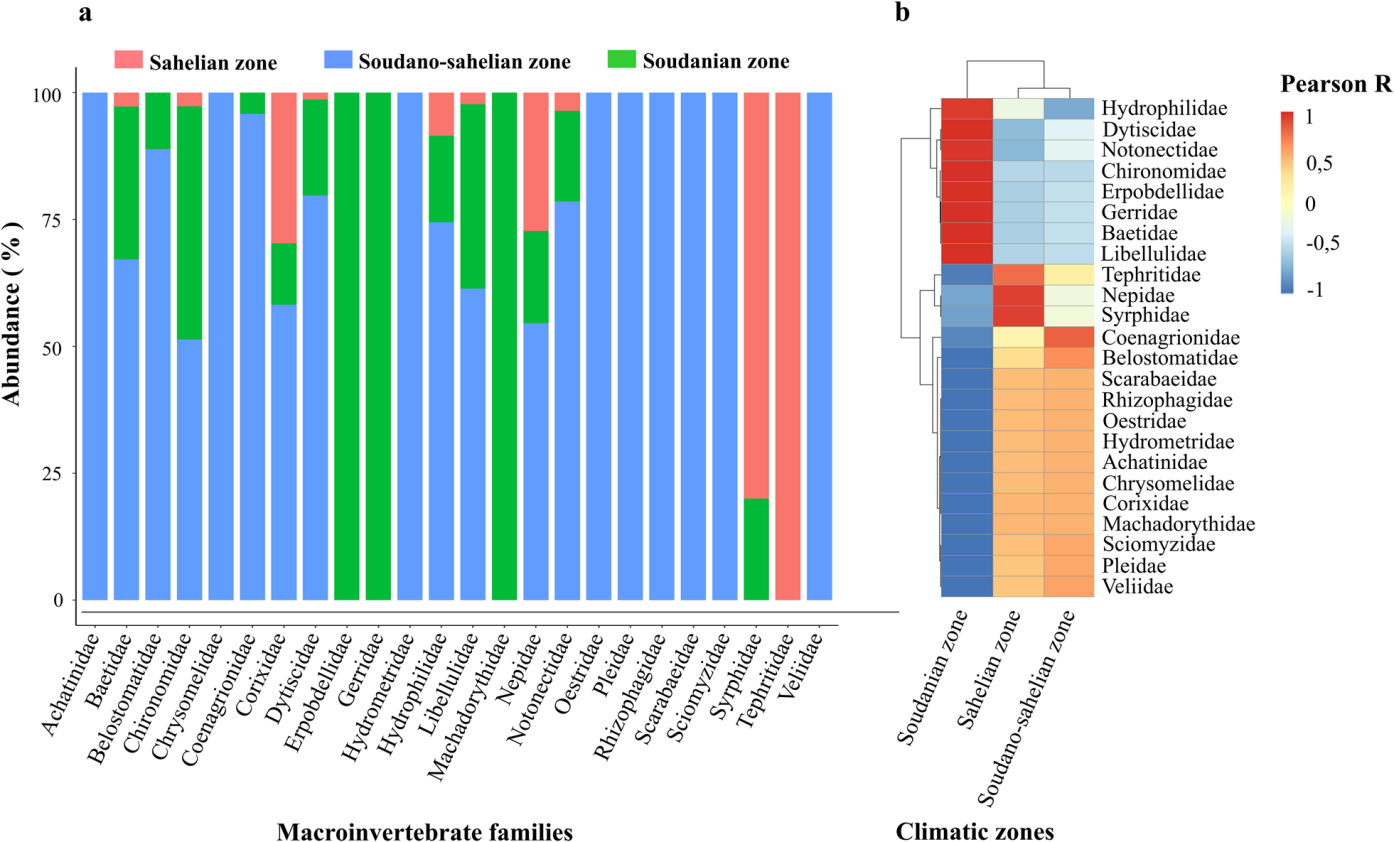
Distribution of predatory macroinvertebrates and other coexisting macroinvertebrates according to climatic zones (**a**), correlation matrix between climatic zones, predatory macroinvertebrates, and other coexisting macroinvertebrates (**b**)

Of the 544 macroinvertebrate predators and other coexisting macroinvertebrates collected, 68 (12.5%) came from the Sahelian zone, 362 (66.5%) from the Soudano-Sahelian zone, and 114 (21%) from the Soudanian zone. According to climatic zones, the abundance of predatory and other coexisting macroinvertebrates collected in different aquatic habitats varied significantly (Kruskal–Wallis H test, *χ*^2^ = 9.20, *df* = 2, *P* = 0.01), with higher abundance in the Soudano-Sahelian region, followed by the Soudanian zone. The Sahelian zone is the climatic zone where macroinvertebrate abundance was the lowest. The abundance of predatory and other coexisting macroinvertebrates according to climatic zones is shown in the Fig. 3a. The correlation matrix between the abundance of these macroinvertebrates and the climatic zones shown that the taxa associated with the Soudanian zone were the Hydrophilidae, Dystiscidae, Chironomidae, Erpobdellidae, Gerridae, Baetidae, and Libellulidae. This study also showed that Nepidae, Syrphidae, and Thephritidae were associated with the Sahelian zone and Coenagrionidae and Belostomatidae were associated with the Soudano-Sahelian zone (Fig. 3b).

### Diversity of predatory and other coexisting macroinvertebrates according to climatic zones

**Fig. 4:**
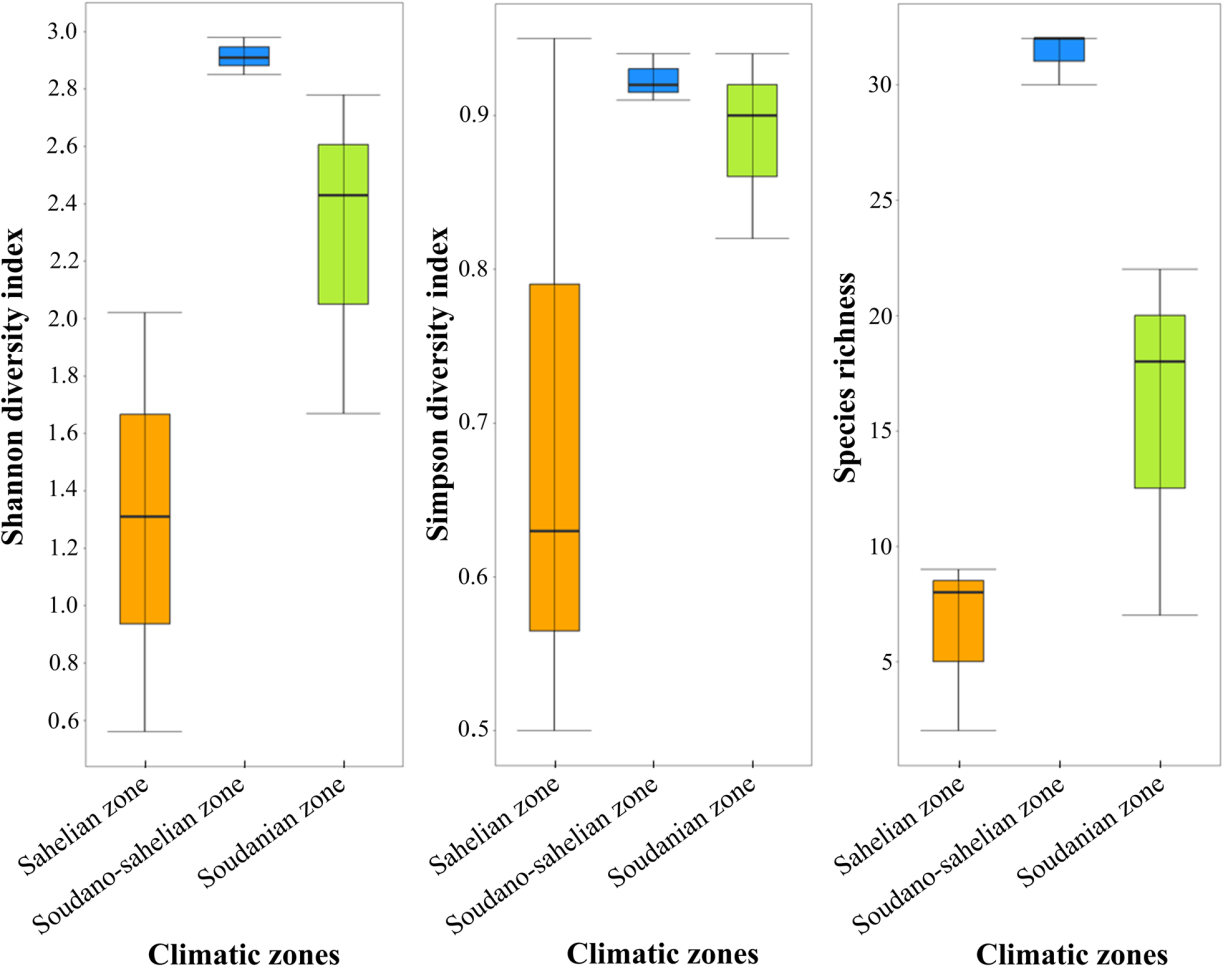
Variation in alpha diversity indices for predatory macroinvertebrates and other coexisting macroinvertebrates associated with *Anopheles* spp. breeding sites collected according to climatic zones

The diversity of macroinvertebrate predators and other coexisting macroinvertebrates associated with *Anopheles* larval habitats varied significantly according to climatic zone [Shannon diversity index (Kruskal–Wallis H test, *χ*^2^ = 6.49, *df* = 2, *P* < 0.05) and Richness specific (Kruskal–Wallis H test, *χ*^2^ = 6.01, *df* = 2, *P* < 0.05)]. However, no significant differences were found between Simpson’s diversity index and the climatic zones. According to climatic zones, the Soudano-Sahelian zone registered the highest alpha diversity index [species richness (30, 32, 32), Shannon diversity index (2.98, 2.91, 2.85), and Simpson diversity index (0.94, 0.92, 0.91)]. The climatic zone that follows the Soudano-Sahelian zone in terms of diversity was the Soudanian zone [species richness (18, 22, 7), Shannon diversity index (2.43, 2.78, 1.67), and Simpson diversity index (0.90, 0.94, 0.82)]. The climatic zone with the lowest alpha diversity index was the Sahelian zone with a specific richness per month of 8, 9, 2. This climatic zone had a Shannon diversity index per month, respectively, of 2.02, 1.31, 0.56, and a Simpson diversity index of 0.95, 0.63, 0.50 (Fig. 4). Tables 2, 3, and 4 list the predatory and other coexisting macroinvertebrates taxa sampled by climatic zone.

**Table 2:** List of macroinvertebrate predators and other coexisting macroinvertebrate taxa associated with *Anopheles* breeding sites in Sahelian zone

Family	Genus	Species number	Species/morphospecies
Chironomidae	*Chironomus*	1	*Chironomus* morphospecies 1
Corixidae	*Micronecta*	3	*Micronecta scutellarus*
			*Micronecta quadristrigata*
			*Micronecta ludibunda*
Dytiscidae	*Hygrotus*	1	*Hygrotus nubilus*
Hydrophilidae	*Berosus*	1	*Berosus pulchellus*
	*Hemiosus*	1	*Hemiosus* morphospecies 1
	*Sternolophus*	1	*Sternolophus rufipes*
Libellulidae	*Libellulidae genus1*	1	*Libellulidae* morphospecies 1
Nepidae	*Nepa*	2	*Nepa* morphospecie 1
			*Nepa* morphospecie 2
Notonectidae	*Anisops*	1	*Anisops* morphospecies 1
Syrphidae	*Eristalis*	1	*Eristalis* morphospecies 1
Tephritidae	*Bactrocera*	1	*Bactrocera* morphospecie 1

**Table 3:** List of macroinvertebrates predators and other coexisting macroinvertebrate taxa associated with *Anopheles* breeding sites in Soudano-Sahelian zone

Family	Genus	Species number	Species/morphospecies
Archatinidae	*Archachatina*	1	*Archacatina* morphospecies 1
Baetidae	*Cloeon*	6	*Cloeon* morphospecies 1
			*Cloeon* morphospecies 2
			*Cloeon* morphospecies 3
			*Cloeon* morphospecies 4
			*Cloeon* morphospecies 5
			*Cloeon* morphospecies 6
Belostomatidae	*Diplonychus*	4	*Diplonychus* morphospecies 1
			*Diplonychus* morphospecies 2
			*Diplonychus* morphospecies 3
			*Diplonychus* morphospecies 4
Chironomidae	*Chironomus*	3	*Chironomus* morphospecies 1
			*Chironomus* morphospecies 2
			*Chironomus* morphospecies 3
Chrysomelidae	*Diabrotica*	1	*Diabrotica* virgifera
Coenagrionidae	*Africallagma*	1	*Africallagma glaucum*
	*Agriocnemis*	1	*Agriocnemis* morphospecies 1
	*Argia*	1	*Argia cupraurea*
	*Enallagma*	1	*Enallagma* morphospecies 1
	*Agrion*	1	*Agrion* morphospecies 1
Corixidae	*Micronecta*	4	*Micronecta scutellaris*
			*Micronecta quadristrigata*
			*Micronecta ludibunda*
			*Micronecta* morphospecies 1
Dytiscidae	*Laccophilus*	3	*Laccophilus continentalis*
			*Laccophilus luteosignatus*
			*Laccophilus enigmaticus*
	*Dytiscus*	1	*Dytiscus* morphospecies 1
	*Canthydrus*	1	*Canthydrus koppi*
	*Liodessus*	2	*Liodessus* morphospecies 1
			*Liodessus* morphospecies 2
	*Hygrotus*	3	*Hygrotus nubilus*
			*Hygrotus* morphospecies 1
			*Hygrotus* morphospecies 2
Hydrometridae	*Hydrometra*		*Hydrometra australis*
Hydrophilidae	*Berosus*	4	*Berosus pulchellus*
			*Berosus* morphospecies 1
			*Berosus* morphospecies 2
			*Berosus* morphospecies 3
	*Hemiosus*	3	*Hemiosus* morphospecies 1
			*Hemiosus* morphospecies 2
			*Hemiosus* morphospecies 3
	*Sternolophus*	1	*Sternolophus* morphospecies 1
	*Laccobius*	1	*Laccobius* morphospecies 1
	*Hydrophilidae* genus 1	1	*Hydrophilidae* morphospecies 1
Libellulidae	*Pantala*	1	*Pantala flavescens*
	*Crocothemis*	1	*Crocothemis nugrifons*
	*Urothemis*	1	*Urothemis thomasis*
	*Orthemis*	1	*Orthemis* morphospecies 1
	*Tholymis*	1	*Tholymis tillarga*
	*Hydrobasileus*	1	*Hydrobasileus brevistylus*
	*Agrionoptera*	1	*Agrionoptera longitudinalis*
	*Libellulidae* genus 1	1	*Libellulidae* morphospecies 1
Nepidae	*Ranatra*	1	*Ranatra gracilis*
Notonectidae	*Enithares*	2	*Enithares* morphospecies 1
			*Enithares* morphospecies 2
	*Anisops*	4	*Anisops* morphospecies 1
			*Anisops* morphospecies 2
			*Anisops* morphospecies 3
			*Anisops* morphospecies 4
	*Notonecta*	1	*Notonecta* morphospecies 1
Oestridae	*Oestridae* genus 1	1	*Oestridae* morphospecies 1
Pleidae	*Paraplea*	1	*Paraplea frontalis*
Rhizophagidae	*Rhizophagus*	1	*Rhizophagus* morphospecies 1
Scarabaeidae	*Ataenuis*	1	*Ataenuis* morphospecies 1
Sciomyzidae	*Hedria*	1	*Hedria* morphospecies 1
Vellidae	*Microvelia*	1	*Microvelia* morphospecies 1

**Table 4:** List of macroinvertebrates predators and other coexisting macroinvertebrate taxa associated with *Anopheles* breeding sites in Soudanian zone

Family	Genus	Species number	Species/morphospecies
Baetidae	*Cloeon*	9	*Cloeon* morphospecies 1
			*Cloeon* morphospecies 2
			*Cloeon* morphospecies 3
			*Cloeon* morphospecies 4
			*Cloeon* morphospecies 5
			*Cloeon* morphospecies 6
			*Cloeon* morphospecies 7
			*Cloeon* morphospecies 8
			*Cloeon* morphospecies 9
Belostomatidae	*Belostoma*	1	*Belostoma* morphospecies 1
Chironomidae	*Polydedilum*	1	*Polydedilum* morphospecies 1
	*Chironomus*	1	*Chironomus* morphospecies 1
Coenagrionidae	*Enallagma*	1	*Enallagma* morphospecies 1
Corixidae	*Micronecta*	4	*Micronecta scutellaris*
			*Micronecta quadristrigata*
			*Micronecta ludibunda*
			*Micronecta* morphospecies 1
Dytiscidae	*Hygrotus*	3	*Hygrotus nubilus*
			*Hygrotus* morphospecies 1
			*Hygrotus* morphospecies 2
	*Laccophilus*	1	*Laccophilus saegeri*
	*Copelatus*	1	*Copelatus* morphospecies 1
Erpobdellidae	*Dina*	1	*Dina lineata*
Gerridae	*Neogerris*	1	*Neogerris* morphospecies 1
	*Tachymetra*	1	*Tachymetra* morphospecies 1
	*Brachymetra*	1	*Brachymetra* morphospecies 1
Hydrophilidae	*Berosus*	2	*Berosus pulchellus*
			*Berosus* morphospecies 1
	*Sternolophus*	1	*Sternolophus rufipes*
Libellulidae	*Pantala*	1	*Pantala flavescens*
	*Urothemis*	1	*Urothemis thomasis*
	*Diplacodes*	1	*Diplacodes* morphospecies 1
	*Crocothemis*	1	*Crocothemis nugrifons*
	*Rhodothemis*	1	*Rhodothemis* morphospecies 1
	*Libellula*	1	*Libellula* morphospecies 1
	*Libellulidae Genus 1*	1	*Libellulidae* morphospecies 1
Machadorythidae	*Machadorythus*	1	*Machadorythus* morphospecies 1
Nepidae	*Nepa*	1	*Nepa* morphospecies 1
Notonectidae	*Anisops*	2	*Anisops* morphospecies 1
			*Anisops* morphospecies 2
Syrphidae	*Eristalis*	1	*Eristalis* morphospecies 1

### Relationship between *Anopheles* larvae, macroinvertebrate predators, and other coexisting macroinvertebrates

**Fig. 5:**
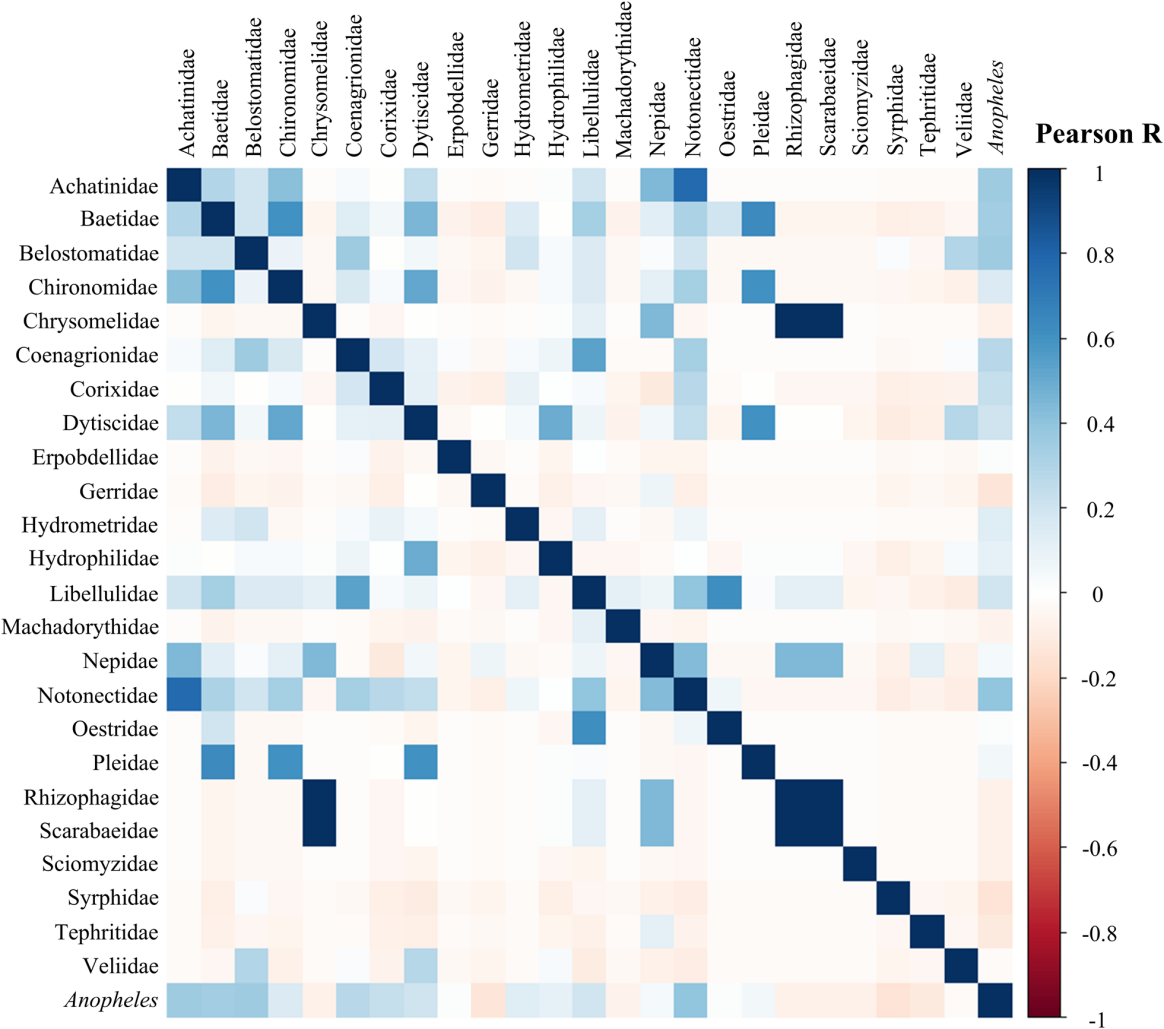
Correlation matrix between *Anopheles* spp. larvae and predatory macroinvertebrates and other coexisting macroinvertebrates

Pearson’s correlation coefficients between *Anopheles*, macroinvertebrate predators, and other coexisting macroinvertebrates were calculated and visualized in a heatmap. In all climatic zones, the analysis showed a weak positive correlation in larval habitats between *Anopheles* spp. larvae and Notonectidae (Pearson’s correlation coefficient, *r* = 0.40, *P* < 0.001), Achatinidae (Pearson’s correlation coefficient, *r* = 0.36, *P* = 0.003), Baetidae (Pearson’s correlation coefficient, *r* = 0.35, *P* = 0.004), and Belostomatidae (Pearson’s correlation coefficient, *r* = 0.35, *P* = 0.004) (Fig. 5).

**Fig. 6:**
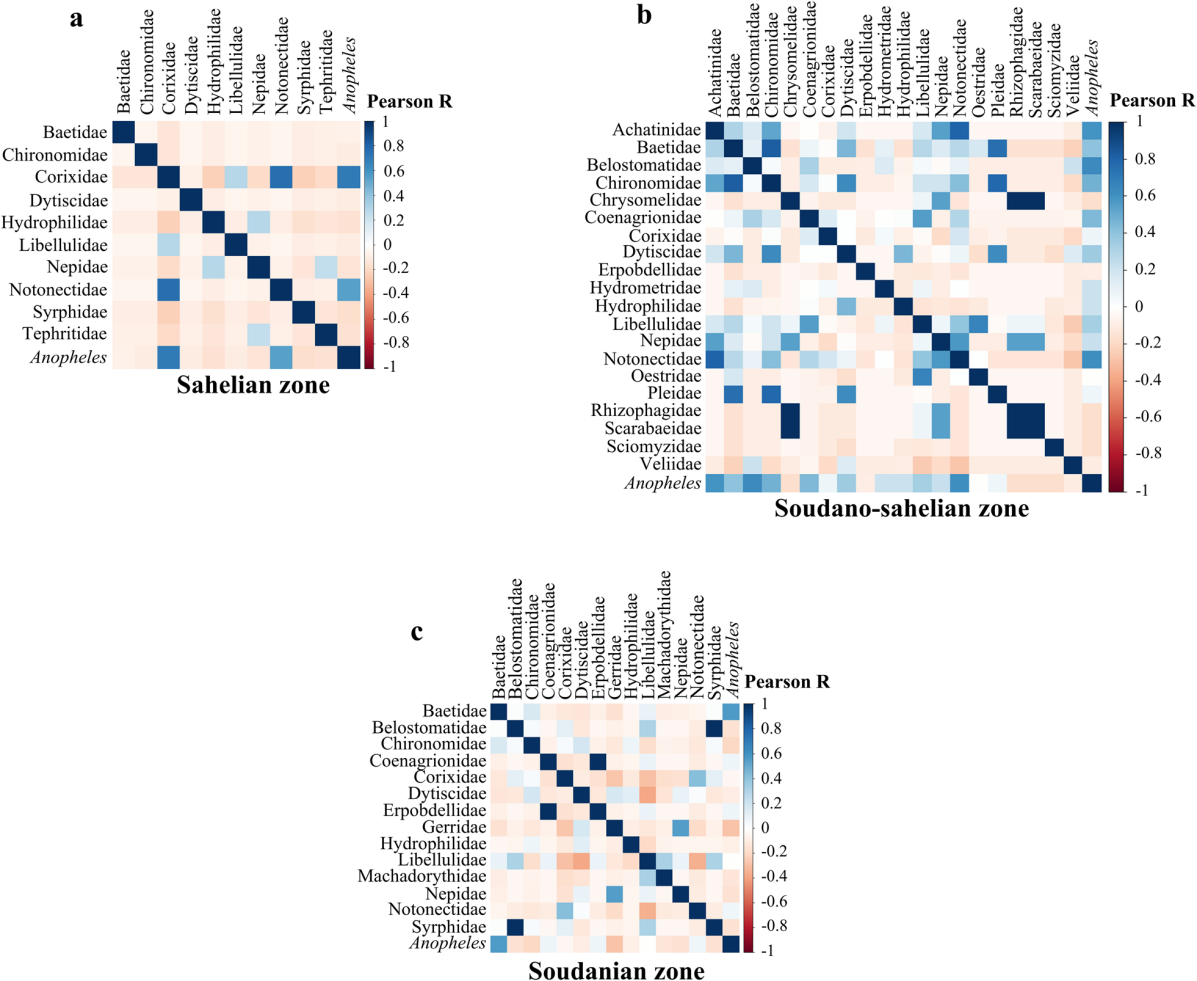
Correlation matrix of groupings in the Sahelian zone (**a**), Soudano-Sahelian zone (**b**), and Soudanian zone (**c**)

In the Sahelian zone, significant positive correlations were found between the abundance of *Anopheles* and those of Corixidae (Pearson’s correlation coefficient, *r* = 0.71, *P* < 0.001) and Notonectidae (Pearson’s correlation coefficient, *r* = 0.54, *P* = 0.02). In the Soudano-Sahelian zone, the abundance of *Anopheles* spp. was positively correlated with the abundance of Achatinidae (Pearson’s correlation coefficient, *r* = 0.59, *P* = 0.005), Belostomatidae (Pearson’s correlation coefficient, *r* = 0.63, *P* = 0.002), and Notonectidae (Pearson’s correlation coefficient, *r* = 0.61, *P* = 0.003). However, only the abundance of Baetidae was positively correlated with *Anopheles* in the Soudanian zone (Fig. 6).

**Fig. 7:**
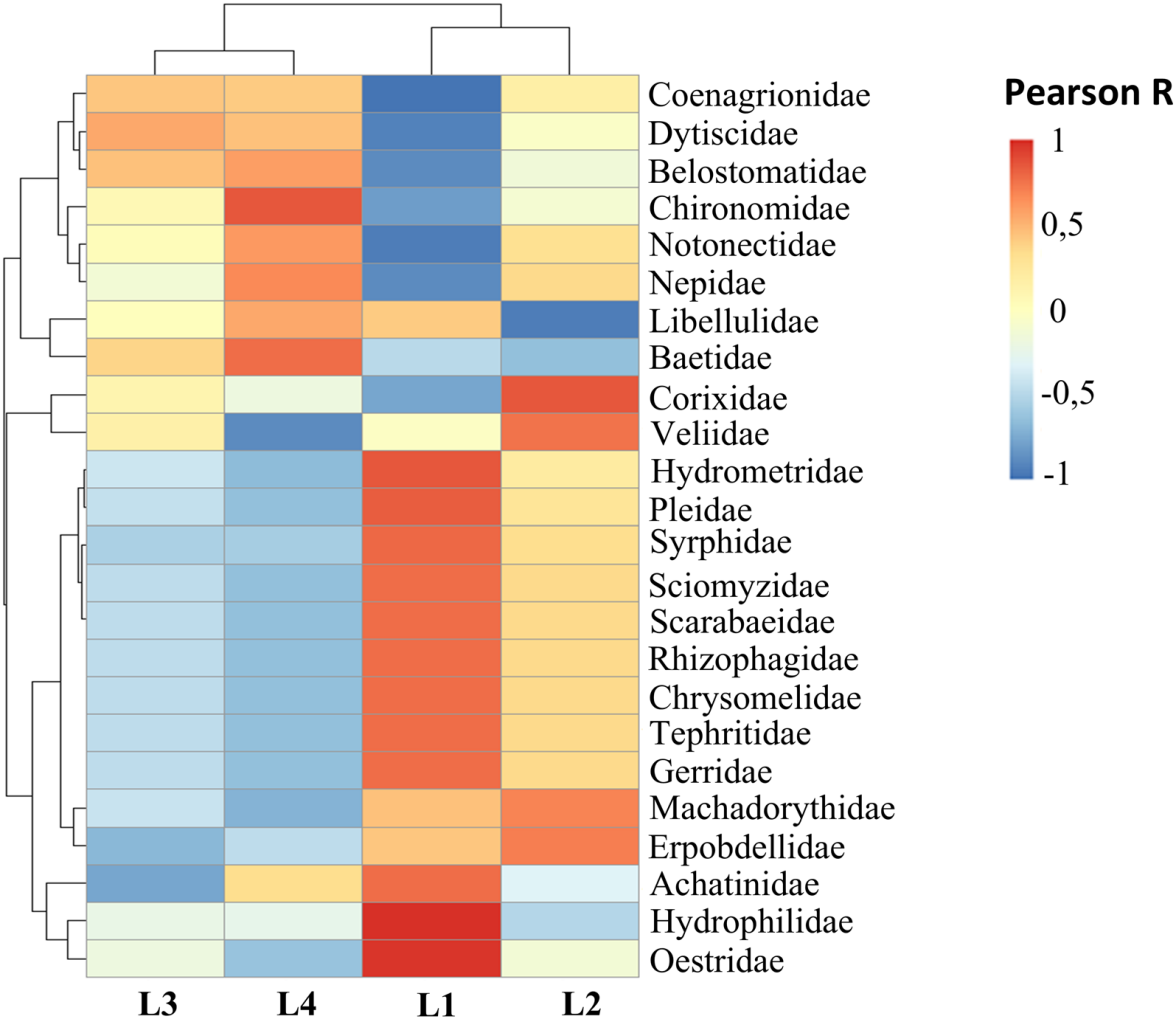
Association between macroinvertebrates and *Anopheles* larval stages in aquatic habitats

In larval habitats, the abundance of macroinvertebrate families was correlated with the abundance of *Anopheles* spp. larval stage. The presence of certain families of macroinvertebrates has influenced the abundance of larval stages (Fig. 7).

## Discussion

Macroinvertebrate predators and other coexisting macroinvertebrates could influence the abundance of *Anopheles gambiae* s.l., the malaria major vector in Burkina Faso, by feeding on their larvae or by competition in sharing resources in aquatic environments [17, 35]. Improving knowledge of interaction between anopheline mosquito and other macroinvertebrates could help to improve biocontrol strategies. Here, various associations between *Anopheles*, macroinvertebrate predators, and other coexisting macroinvertebrates in larval habitats were investigated. We also determined the distribution of macroinvertebrates according to climate, and shed light on the different associations existing between the *Anopheles* larval stages, macroinvertebrate predators, and coexisting macroinvertebrates in larval habitats.

In larval habitats sampled in this study, 24 families of macroinvertebrates cohabiting with *Anopheles* were identified. Certain families of macroinvertebrates, such as Corixidae, Dytiscidae, Hydrophilidae, Libellulidae, Notonectidae, Coenagrionidae, Nepidae, and Belostomatidae characterized in this study are known to feed on anopheline mosquito larvae[1, 36–38].These predators could contribute to the bioregulation of malaria vector populations and control this disease. Other nonpredatory macroinvertebrates could also have competitive interactions with *Anopheles* larvae through sharing of resources and indirect interactions. Previous studies have shown that *Anopheles* larvae cohabit with macroinvertebrate predators and other coexisting macroinvertabrates in larval habitats [17, 18, 30, 39]. A study performed in Uganda shows that macroinvertebrates such as Dytiscidae, Notonectidae, Baetidae, Coenagrionidae, Aeshnidae, Haliplidae, and Elmidae have been found in aquatic habitats such as ponds, streams, temporary pools, and roadside ditches [17]. In Burkina Faso, in the study performed by Diabaté et al. [18] in the Kou Valley (Bama), a rice growing area, the macroinvertebrate families characterized were Notonectidae, Dystiscidae, Corixidae, Hydrophilidae, and Libellulidae.

Spatial distribution of macroinvertebrates varied significantly according to climatic zones. Data in this study suggest that, depending on the climatic zone, there are macroinvertebrate predators that contribute to the biocontrol of mosquito populations. Although studies have shown that the majority of macroinvertebrate predators families characterized are highly effective predators against mosquito larvae [1, 40], pollution of larval habitats by pesticide residues threatens the effectiveness of its predators. Several studies have linked pesticide pollution of larval habitats to a reduction in the macroinvertebrate fraction [41, 42]. Insecticides can cause the direct mortality of the natural enemies of *Anopheles* larvae [43, 44]. Measures must be taken to prevent the threat of pollution of breeding sites by pesticides commonly used in agriculture to conserve and improve the biodiversity of these predators.

In this study, the highest diversity was observed in the Soudano-Sahelian zone. The highest abundance and diversity of macroinvertebrates found in the Soudano-Sahelian zone is thought to be related to the long period of retention of water in larval habitats due to average rainfall, which prevents leaching and drying out of larval habitats, compared with the Soudanian zone with abundant rainfall. Furthermore, in the Sahelian zone, the low abundance and diversity found would be linked to low rainfall, which favors the drying out of larval habitats, making survival conditions difficult for predatory and other coexisting macroinvertebrates. Other studies have suggested that permanent larval habitats provide favorable conditions for macroinvertebrate predators and other coexisting macroinvertebrates as previously shown by Bonds et al., Link et al., and Egler et al. [45–47].

Overall, in all climatic zones, no significant association was found between *Anopheles* larvae abundance and the other macroinvertebrates abundance sampled in larval habitats. However, depending on the climatic zone, certain macroinvertebrate families were strongly correlated with *Anopheles* larvae in larval habitats. Predators can consume *Anopheles* larvae, reducing their survival and population size, and this association between anopheline mosquito larvae and the predatory and other coexisting macroinvertebrates can be explained by the fact that some larval predators have developed a behavior of detecting *Anopheles* larval habitats. In Uganda, *Anopheles gambiae* s.l. larvae have been shown to cohabit with predators such as Dytiscidae and Cybaeidae [17]. Previous studies documented predation behavior of some predators against anopheline larval [48], and several families of aquatic predators have been shown to be effective in reducing mosquito survival in terms of consuming *Anopheles* larvae [30]. Testing aquatic macroinvertebrates commonly found in Burkina Faso could help to identify previously unknown predators.

We also show in this study that there are correlations between *Anopheles* larval stages and certain macroinvertebrate predators and other coexisting macroinvertebrates in aquatic habitats. This finding could be explained by the fact that these macroinvertebrate predators are specialized in consuming a specific anopheline mosquito larval stage, resulting in a reduction in the specific larval stage consumed to the detriment of other stages or through the development of behavior of avoiding larval habitats containing certain predators by females in search of egg-laying sites. Studies have reported that the consumption of mosquito larvae by predators depends on the larval stage [1]. The difficulty for bioregulation-based management of anopheline larvae will be to optimize predator composition by covering all larval stages. More studies on predation efficiency on different life-stages is recommended.

In addition to consumption effects, predators can also have non-consumption effects on *Anopheles* characteristics. They may have an impact on mosquito body size and survival through non-consumptive effects [49]. These results show that the association between macroinvertebrate predators and mosquito larvae in larval habitats has implications for malaria control, as the biological control of mosquito larvae through the use of macroinvertebrate predators could be a cost-effective and easily applicable strategy [50]. However, predation efficiency, macroinvertebrate enrichment, and life stage-specific and sublethal effects of predation on *Anopheles* merit definitely further research.

One of the limitations of this study is that it did not investigate the impact of residual pesticides used in agriculture on predators and *Anopheles* spp. larvae in larval habitats. It is therefore necessary to understand the impact of pesticides used in agriculture on predators. Presence of pesticide residues in larval habitats can cause predator mortality or reduce their effectiveness in controlling vector populations. Further investigations should focus on the impact of pesticide residues and physicochemical parameters of the water in the larval habitats sampled, such as temperature, pH, and water conductivity, which could influence the spatiotemporal distribution of macroinvertebrates. Although this does not affect our interpretation of the results, it would be also interesting to collect data during the dry and rainy seasons to better understand the effects of seasonal variation on macroinvertebrate diversity and predator–prey interactions.

## Conclusions

This study showed evidence of the existence of a diversity of macroinvertebrates that could play a predatory role on *Anopheles* larvae in larval habitats in Burkina Faso. More than 24 families of predatory and other coexisting macroinvertebrates were identified and their abundance varied according to climatic zone. The presence of certain families of macroinvertebrates in the larval habitats has a significant effect on the abundance of *Anopheles* spp., demonstrating the possibility of using them for larval control. Our next objective is to assess the predatory efficiency of commonly cohabiting macroinvertebrates commonly found in Burkina Faso.

## Data Availability

The data used in this article are available on request by contacting the corresponding authors.

## Abbreviations

pH: Potential hydrogen
s.l.: Sensu lato
spp.: Multiple species of the genus
C: Degrees Celsius
